# Supplementary figures and images for: A recurrent neural network and parallel hidden Markov model algorithm to segment and detect heart murmurs in phonocardiograms
Source: PLOS Digit Health. 2024 Nov 25;3(11):e0000436. doi: 10.1371/journal.pdig.0000436 (PMC11588198; doi:10.1371/journal.pdig.0000436)

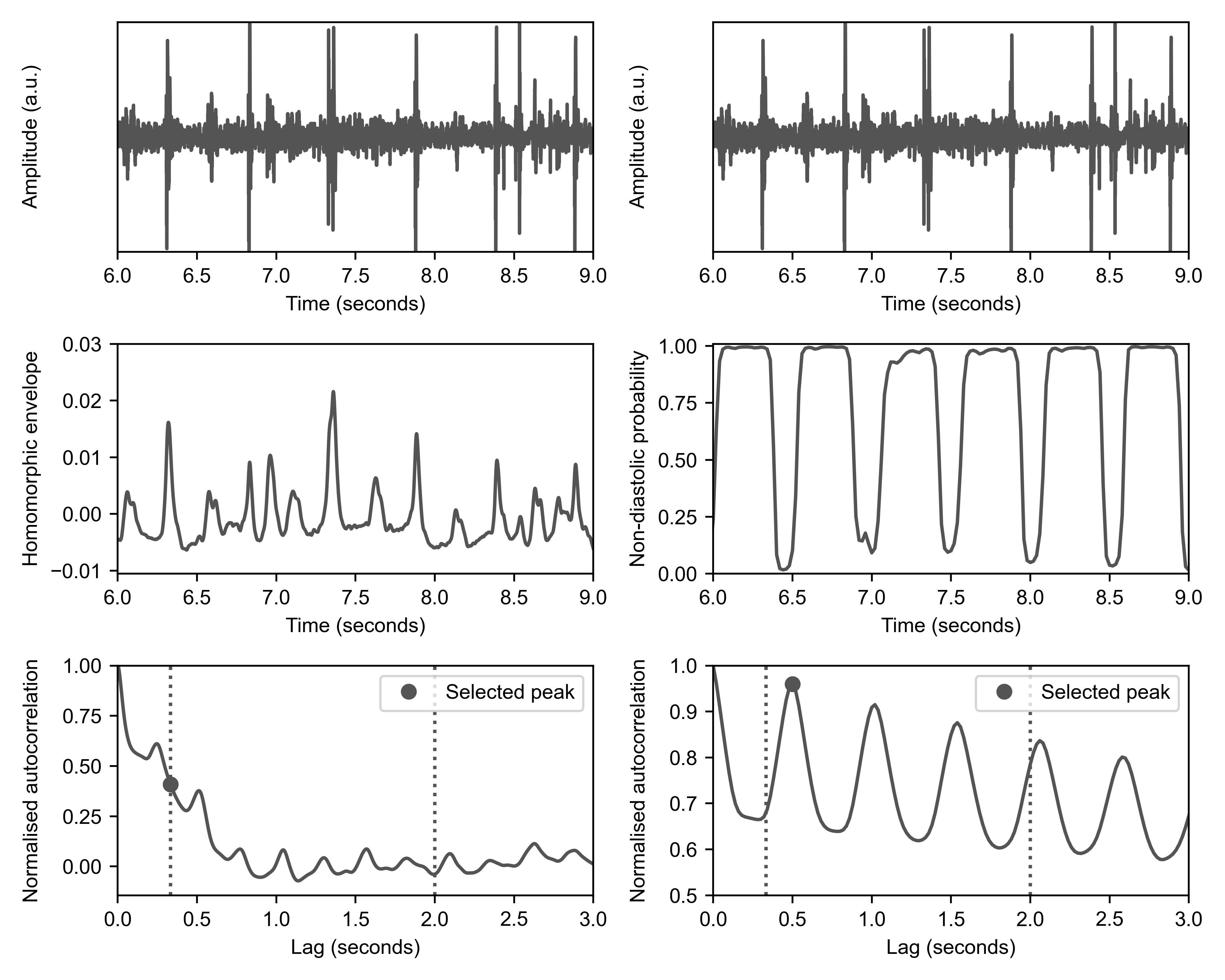

Supplement: S1 Fig — Compared here are the methods of Schmidt et al. [23] (left column) and our approach (right column). Schmidt et al. take a homomorphic envelope (middle left) of the signal and then compute its autocorrelation (bottom left). They then search for a peak in a specified range to estimate the heart rate. We use a range of 30-180 bpm for both methods in this dataset because of the faster paediatric sounds. However, this example heart sound (top left) has significant noise which corrupts the envelope and therefore gives a noisy autocorrelation where the correct peak is difficult to find. Our approach instead uses the output of the RNN to create a signal that shows the probability the signal is not in diastole, P(qt ≠ diastole|x1:T, θ), (i.e. the summed probability of the S1, S2, systole, and systolic murmur states, middle right). This is a much cleaner signal than the homomorphic envelope, so its autocorrelation (bottom right) is much clearer and the correct peak corresponding to the signal period is easy to find. (TIF) [file pdig.0000436.s001.tif]
